# Control of Mooij correlations at the nanoscale in the disordered metallic Ta–nanoisland FeNi multilayers

**DOI:** 10.1038/s41598-020-78185-6

**Published:** 2020-12-03

**Authors:** N. N. Kovaleva, F. V. Kusmartsev, A. B. Mekhiya, I. N. Trunkin, D. Chvostova, A. B. Davydov, L. N. Oveshnikov, O. Pacherova, I. A. Sherstnev, A. Kusmartseva, K. I. Kugel, A. Dejneka, F. A. Pudonin, Y. Luo, B. A. Aronzon

**Affiliations:** 1 Department of Physics, Loughborough University, Loughborough, LE11 3TU UK; 2 P.N. Lebedev Physical Institute, Russian Academy of Sciences, Moscow, 119991 Russia; 3 Micro/Nano Fabrication Laboratory (MNFL), Microsystem and Terahertz Research Center, Chengdu, 610200 China; 4 National Research Center, Kurchatov Institute, Moscow, 123182 Russia; 5 Institute of Physics, Academy of Sciences of the Czech Republic, Prague, 18221 Czech Republic; 6 Institute for Theoretical and Applied Electrodynamics, Russian Academy of Sciences, Moscow, 125412 Russia; 7 National Research University Higher School of Economics, Moscow, 101000 Russia

**Keywords:** Materials science, Nanoscience and technology, Optics and photonics, Physics

## Abstract

Localisation phenomena in highly disordered metals close to the extreme conditions determined by the Mott-Ioffe-Regel (MIR) limit when the electron mean free path is approximately equal to the interatomic distance is a challenging problem. Here, to shed light on these localisation phenomena, we studied the dc transport and optical conductivity properties of nanoscaled multilayered films composed of disordered metallic Ta and magnetic FeNi nanoisland layers, where ferromagnetic FeNi nanoislands have giant magnetic moments of 10$^3$–10$^5$ Bohr magnetons ($\mu _{\mathrm{B}}$). In these multilayered structures, FeNi nanoisland giant magnetic moments are interacting due to the indirect exchange forces acting via the Ta electron subsystem. We discovered that the localisation phenomena in the disordered Ta layer lead to a decrease in the Drude contribution of free charge carriers and the appearance of the low-energy electronic excitations in the 1–2 eV spectral range characteristic of electronic correlations, which may accompany the formation of electronic inhomogeneities. From the consistent results of the dc transport and optical studies we found that with an increase in the FeNi layer thickness across the percolation threshold evolution from the superferromagnetic to ferromagnetic behaviour within the FeNi layer leads to the delocalisation of Ta electrons from the associated localised electronic states. On the contrary, we discovered that when the FeNi layer is discontinuous and represented by randomly distributed superparamagnetic FeNi nanoislands, the Ta layer normalized dc conductivity falls down below the MIR limit by about 60%. The discovered effect leading to the dc conductivity fall below the MIR limit can be associated with non-ergodicity and purely quantum (many-body) localisation phenomena, which need to be challenged further.

## Introduction

The phenomenon of negative temperature coefficient of resistivity (TCR) characterising linearly temperature-dependent resistivity variation $\rho (T)=\rho _0\left[ 1+\alpha _0(T-T_0) \right]$ is found in many highly disordered metals, amorphous metals, and metallic glasses in the form of both bulk solids and thin films. There the TCR ($\alpha_0$) values tend to be more negative with increasing resistivity $\rho _0$ as demonstrated by the numerous data collected in the original Mooij plot^1^. This phenomenon is beyond the ordinary Boltzmann theory of transport in metals, which rigorously requires that $d\rho /dT>0$. The original Mooij plot indicates that TCR changes sign in a relatively narrow range of resistivities $\rho _c$ $\simeq$ 100–150  $\mu \Omega \cdot$cm. However, subsequently, it was argued by Tsuei that the original Mooij plot ($\alpha _0$ vs $\rho _0$) is not universal^2^. It was shown that for the three-dimensional (3D) case $\rho _c$ depends on the material characteristics, Fermi surface wave vector $k_{\mathrm{F}}$ and the elastic mean free path $l_e$ of conduction electrons. More than 500 data points were collected from the literature by Tsuei in his seminal work^2^ indicating that $\rho _c$ spans from $\sim$ 30 to $\sim$ 300 $\mu \Omega \cdot$cm in the Mooij plot coordinates.

Different microscopic scenarios have been developed to explain the origin of Mooij correlations in disordered metals. Following the chronological sequence, earlier the TCR and $\rho (T)$ were fairly well described using the physical concepts that have been put forward for liquid metals^3^. Some of them (for example, Hg and liquid Te) were proposed by Mott^4^ as candidates for a metal at the borderline of the nonconducting behavior. Indeed, they demonstrate an intriguing behaviour where optical conductivity $\sigma _1(\omega )$ displays a minimum at zero frequency accompanied by a transfer of the oscillator strength to higher frequencies in the form of an infrared peak^5^. Later on, the theory developed for liquid metals was extended for metallic glasses^6,7^. A principal possibility of using the latter approach is based on the suggestion that the elements of the crystal structure are preserved in liquid and amorphous metals. The change in the atomic static structure factor characteristic of the liquid as a function of temperature was calculated including the usual Debye–Waller factor and the phonon contribution.

One popular opinion on the possible cause for the negative TCR phenomenon^2,8–11^ invokes the “weak localisation scenario” and the formation of the impurity-induced bound electronic state, viz., the so-called Anderson localisation^12^. The related quantum-interference processes, or weak localisation corrections^13,14^, provide a consistent theory for the description of the low-temperature dc transport in metals at weak disorder. The “weak localisation” scenario does not assume any significant rearrangement and gap opening in the electronic spectra. In this case, the dc transport will be determined by the competition between the degradation of the quantum-interference effects as a result of inelastic scattering and the conventional Boltzmann electron transport in metals. Metals and alloys remain metallic with the resistivity lower than the critical $\rho ^*$ value corresponding to the Mott-Ioffe-Regel (MIR) limit^15–17^

1 $$\begin{aligned} \rho ^*=\frac{\hbar k_{\mathrm{F}}}{ne^2l_e}=\frac{\hbar }{e^2}\frac{1}{k_{\mathrm{F}}}\sim 300\,\mu \Omega \cdot \mathrm {cm}, \end{aligned}$$

where $\hbar$ is the Planck’s constant. The universality of the Mooji correlations found in highly disordered metals, which suggests an approximately linear correspondence between TCR and resistivity $\rho _0$ ($\alpha _0$ vs $\rho _0$), where $\rho _0$ is referred to the room temperature conditions^1^, is striking. The Mooij correlations found in the high-temperature regime where incoherent thermal phonon excitations abound seems to be highly improbable. However, since under the condition of increased disorder in high-resistivity alloys scattering by static structural defects may occur more frequently than inelastic scattering by phonons, long-distance phase coherence required for interference processes may persist in the appropriate temperature range^2,8–11,18^.

The most recently developed model, which is unrelated to the Anderson localisation mechanism, was suggested by Ciuchi et al.^19^. The origin of the Mooij correlations is associated with a strong enhancement of polaronic effects due to disorder^19,20^. It is suggested that the reduced mobility of electrons in poor metallic conductors may allow the lattice deformations to self-trap electrons through a disorder-assisted polaronic effect^21^. The mechanism may acquire a dominant role at sufficiently strong disorder leading to gap formation and substantial transfer of spectral weight (SW) away from the Fermi energy to the renormalised polaronic states.

The presented above theoretical models questioning the origin of Mooij correlations consider averaged characteristics which may be accurate in description of only homogeneous systems. However, inhomogeneities may be peculiar for disordered metallic systems. For example, the formation of additional defects under irradiation of an amorphous $\hbox {Pd}_{{80}}\hbox {Si}_{{20}}$ alloy with fast neutrons may lead to the formation of inhomogeneous structure, consisting of clusters 10–20 Å in diameter with an enhanced electronic density surrounded by neighbouring regions with devastated electronic density^22^. In addition, in the limit of strong localisation conditions when electronic wave functions become disturbed practically at each atomic site, strong electronic correlations should be taken into account. In general, the formation of electronic inhomogeneities should necessarily lead to the rearrangement of optical SW due to electronic correlations. The situation may resemble that discovered in the Kondo-lattice metal $\hbox {Tb}_2\hbox {PdSi}_3$, where the formation of electronic inhomogeneities^23^ manifests itself in the optical conductivity with opening of the pseudogap and the appearance of the Mott–Hubbard-like electronic excitations capturing the shifted optical SW (see^24^ and the theoretical model presented in the Supplementary online information to the paper^24^). Here electronic correlations inevitably existing between localised electrons^25–28^ may develop when metallic charge carriers become self-localised in the metallic magnetic clusters near the large localised moments of Tb 4f states. This is also supported by the recent evidence of the formation of intrinsic inhomogeneous states represented by ferromagnetic clusters (called magnetic polarons) in magnetic materials, in which the electronic and magnetic properties are strongly modified by the exchange coupling between the conduction electrons and local magnetic moments^29^.

Recently, by using dc transport and wide-band spectroscopic ellipsometry experimental techniques we investigated disordered metallic $\beta$-Ta films^30,31^, which are known to manifest negative TCR^32^. We discovered that with increasing degree of disorder the Drude contribution due to intraband absorption within the Ta 5d $t_{2g}$ band at the Fermi level decreases and simultaneously the higher-energy optical bands appear as satellites at around 2–4 eV, where the associated optical SW is recovered. The discovered optical SW transfer can hardly be associated with the mechanism proposed for liquid metals by Smith^5^, because the spectral positions of the bands by far exceed the infrared frequencies, rather being characteristic for the systems with strong electron correlations^25–27^.

**Figure 1:**
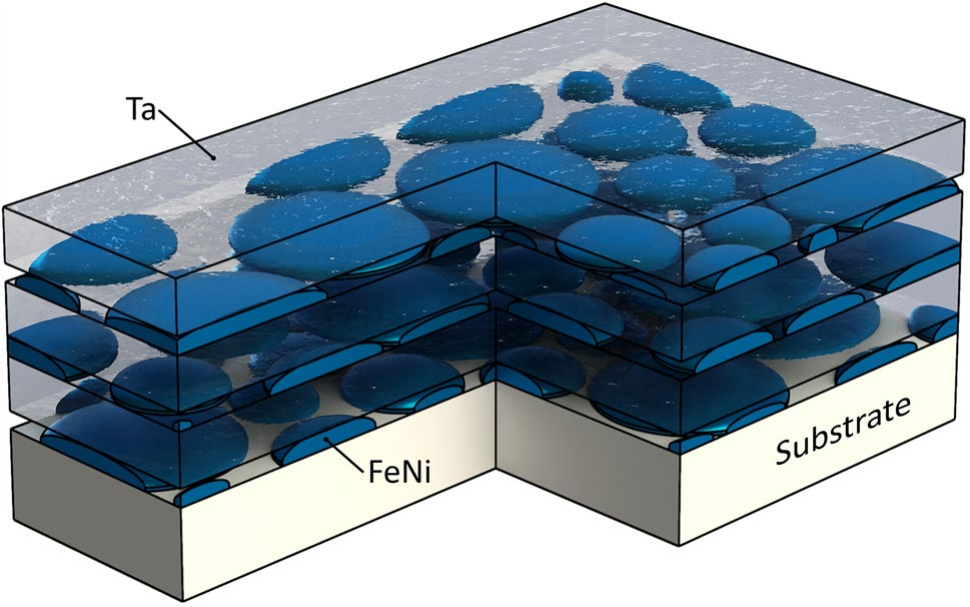
A schematic picture of the multilayer film (MLF) samples (Ta–FeNi)$_{\mathrm{N}}$/Sitall substrate including nanoiland FeNi layers.

To prove or disprove the existence of these phenomena, which can explain the origin of the Mooij correlations associated with the existence of nanoscale electronic inhomogeneities in highly disordered metals, one has to understand how to control them (disturb or disrupt). In the giant magnetoresistance (GMR) layered nanostructures, the interlayer coupling between two ferromagnets is driven by the Ruderman-Kasuya-Kittel-Yoshida- (RKKY-) type^33–35^ indirect exchange interactions via itinerant charge carriers of a nonmagnetic spacer. Having this in mind, here we propose to apply the elaborated approach^30,31^ to study the dc transport and optical conductivity properties of the disordered metallic Ta interlayer in ultrathin multilayer structures Ta–$\hbox {Fe}_{{21}}\hbox {Ni}_{{79}}$ similar to those exhibiting the GMR effect. We investigated the rf-sputtered multilayer films (MLFs) (Ta–FeNi)$_{\mathrm{N}}$ in two limits of the FeNi layer thickness (i) when it is discontinuous and consists of inhomogeneously distributed single-domain ferromagnetic nanoislands having lateral sizes of 5–30 nm^36^ and possessing giant magnetic moments of 10$^3$–10$^5$ $\mu _{\mathrm{B}}$ (where $\mu _{\mathrm{B}}$ is the Bohr magneton) and (ii) when its nominal thickness is varied across the FeNi film percolation threshold around 1.5–1.8 nm^37,38^ (see the schematic picture of the MLF structure (Ta–FeNi)$_{\mathrm{N}}$ involving the nanoisland FeNi layer in Fig. 1). Here, with increasing the FeNi layer thickness across the percolation threshold, the evolution from superparamagnetic (SPM) through superferromagnetic (SFM) to ferromagnetic (FM) behaviour within the FeNi layer^39–41^ will modify the strength of the indirect exchange interaction between neighbouring FeNi layers. This, in turn, will strongly influence the itinerant charge carriers of the Ta layer promoting conditions for their enhanced localisation or delocalisation. It should be mentioned that, in addition to the intrinsic disorder of the Ta layer, its electron transport could strongly be affected by structural and magnetic inhomogeneities of nearby FeNi layers as it can give rise to a large-scale fluctuating potential of electrostatic and/or magnetic origin^42,43^, leading to the appearance of topologically non-trivial spin structures^44^. Moreover, long-range many-body interactions between giant magnetic moments of inhomogeneously distributed FM FeNi nanoislands via itinerant charge carriers of the disordered metallic Ta layer by means of RKKY-type indirect exchange could give rise to a substantial slowing down of all relaxation processes and to the effects of non-ergodicity and purely quantum (many-body) localisation effects^45–49^. Indeed, we discovered that when the FeNi layer is discontinuous and represented by randomly distributed distant FM FeNi nanoislands having giant magnetic moments, the Ta layer dc conductivity falls down below the MIR limit by about 60% (normalised to the MIR limit value). The discovered phenomenon leading to the dc conductivity fall below the MIR limit need to be challenged further. The results of the present study could be important for probing the fundamental physics associated with the localisation phenomena in disordered metals and from the application point of view of the GMR effect.

**Figure 2:**
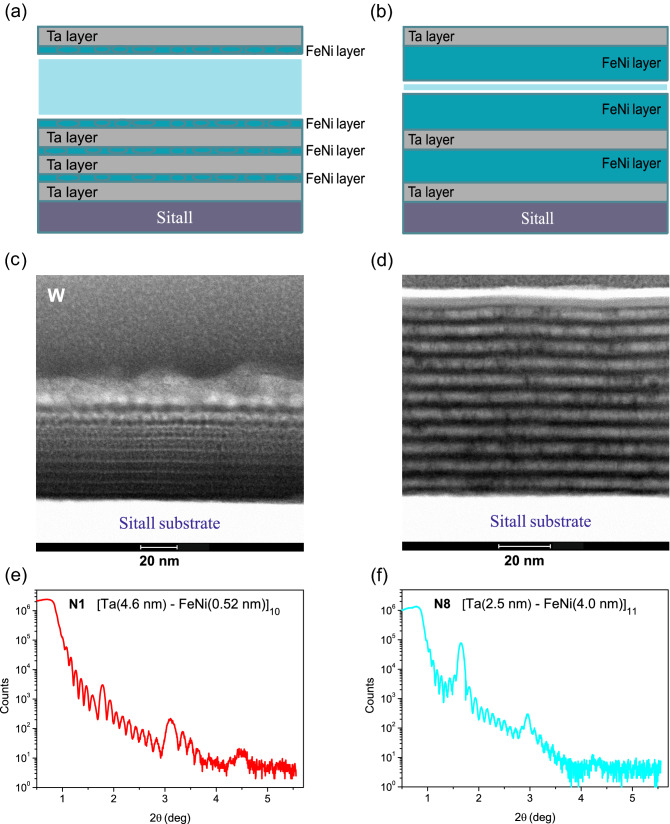
STEM and X-ray reflectivity characterisation of the (Ta–FeNi)_N_ MLF samples. (**a**) For the first series of the MLFs, nominal thickness of the FeNi nanoisland layer was of 0.52 nm and the thickness of the Ta layer varied in the studied samples N1 (4.6 nm), N2 (2.3 nm), and N3 (1.2 nm), where the number of periods was N = 10, 11, and 14, respectively. (**b**) For the second series of the MLFs, the Ta layer thickness was 2.5 nm and the thickness of the FeNi layer varied in the studied samples N5 (1.0 nm), N6 (1.6 nm), N7 (2.0 nm), and N8 (4.0 nm), where the number of periods was N = 11. (**c**,**d**) Bright-field STEM image from the Ta (dark gray colour) and FeNi (light gray colour) layers, and (**e**,**f**) X-ray reflectivity of the MLF samples N1 and N8, respectively.

The MLFs (Ta–FeNi)$_{\mathrm{N}}$–Ta were grown by alternating rf sputtering from 99.95% pure Ta and $\hbox {Fe}_{{21}}\hbox {Ni}_{{79}}$ targets onto insulating Sitall-glass substrates (for more details, see “Methods”). Two series of the MLF samples were prepared. In the first series of the grown (Ta–FeNi)$_{\mathrm{N}}$–Ta/Sitall film samples (hereinafter referred to as N1, N2, and N3), the nominal thickness of the FeNi layer was 0.52 nm; the thickness of the Ta layer was of 4.6, 2.3, and 1.3 nm; and the number of layers was N = 10, 11, and 14, respectively (as schematically shown in Fig. 2a). Here the thickness of the FeNi layer was chosen to be 0.52 nm, when the layer is discontinuous and represented by single-domain FM nanoislands^37^. In the second series of the grown (Ta–FeNi)$_{\mathrm{N}}$–Ta/Sitall film samples (hereinafter referred to as N5, N6, N7, and N8, shown by schematics in Fig. 2b), the number of layers was N = 11, the Ta layer thickness was fixed at 2.5 nm, and the FeNi layer thickness was 1.0, 1.5, 2.0, and 4.0 nm, respectively, increasing above the FeNi film percolation threshold around 1.5–1.8 nm^37,38^. Figure 2c,d shows bright-field scanning/transmission electron microscopy (STEM) images from the Ta and FeNi layers in the cross-section of the MLF samples N1 and N8. The nominal Ta and FeNi thickness values are in good agreement with their values estimated from the cross-section STEM images. One can see that the STEM images reveal well defined interfaces in the MLF structures. The X-ray reflection from the representative MLF samples from the first and second series (N1 and N8) exhibits superlattice peaks (Kiessig’s oscillations), as one can see from Fig. 2e,f, respectively, indicating a periodic compositional modulation along the film growth direction. The observed Kiessig’s oscillations resolved up to the third order give evidence of the relatively small interface roughness (see more details about the MLFs characterisation in the Supplementary information for this paper).

## Results

### Temperature-dependent dc transport of the Ta–FeNi multilayer films

**Figure 3:**
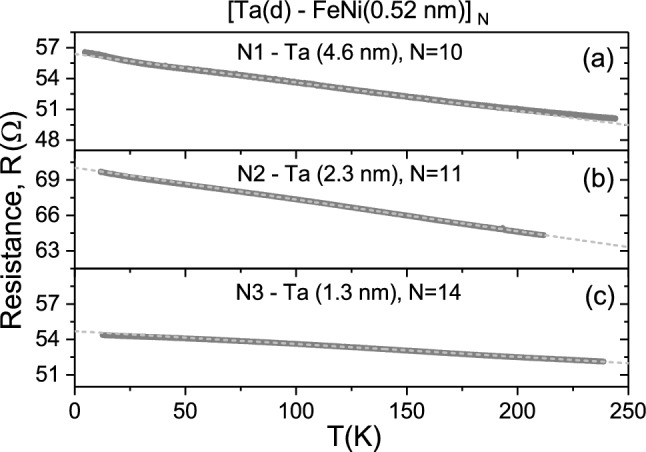
I. Temperature-dependent dc transport for the Ta–FeNi MLFs. (**a**–**c**) The dc resistance *R* of the MLF samples N1–N3, respectively (see Fig. 2a,c,e), approximated by the linear dependence in the 80–180 K temperature range, represented by the dashed lines.

**Figure 4:**
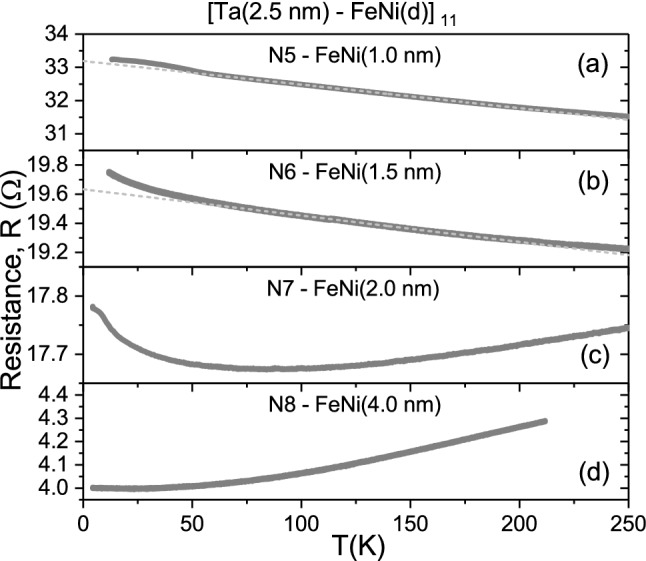
II. Temperature-dependent dc transport for the Ta–FeNi MLFs. (**a**–**d**) The dc resistance *R* of the MLF samples N5–N8, respectively (see Fig. 2b,d,f), approximated by the linear dependence for the MLF samples N5 and N6 in the 80–180 K temperature range, as indicated by the dashed lines.

Figures 3a–c and 4a–d show the temperature dependence of the dc resistance of the MLF samples N1–N3 and N5–N8 measured on heating from the low temperature of 5 K to about 200–250 K. From Fig. 3a–c one can see that the dc resistance of the MLF samples N1, N2, and N3 decreases by about 7–15% with increasing temperature demonstrating non-metallic ($d R/dT<0$) character. We found that in the 80–180 K temperature range the MLFs resistance variation can be well approximated by the linear temperature dependence characterised by the negative slope values, as it is shown in Fig. 3a–c.

**Figure 5:**
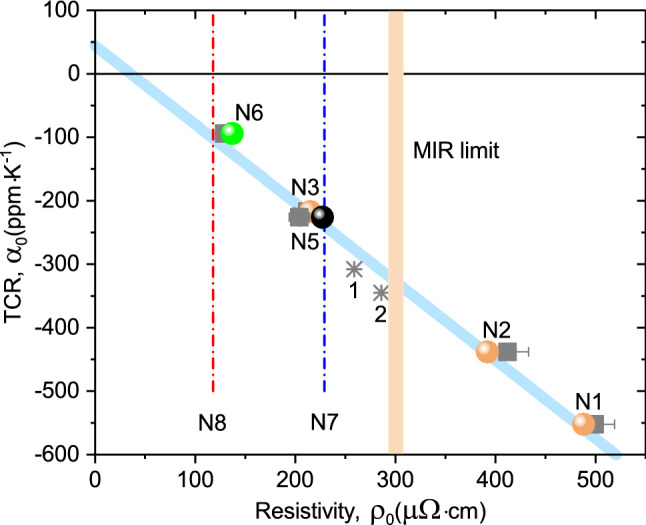
Mooij correlations ($\alpha _0$ vs $\rho _0$) for the Ta layer in the Ta–FeNi MLFs from the dc transport and optical studies. The results of the dc transport study ($\alpha _0$ vs $\rho _0$) for (i) the MLF samples N1–N3, N5, and N6 are represented by square symbols (see also Table 1), and for (ii) the 50- and 33-nm thick single-layer Ta films are shown by asterisks marked as 1 and 2, respectively^31^. The results of the optical study are represented by ($\alpha _0$ vs 1/$\sigma _{1(\omega \rightarrow 0)}$) and include the optical Drude dc limit resistivities (1/$\sigma _{1(\omega \rightarrow 0)}$) for the Ta layer in the MLFs N1–N3 (orange circles)^50^ and N5 (black circle) and N6 (green circle) (see also Fig. 8a,b and Table 2). The dash-dotted lines correspond to the optical Drude dc limit resistivities of the Ta layer in the MLFs N7 and N8, where the $\alpha _0$ values were not possible to extract from the present dc transport measurements (see Eq. (2) and the text). Notice good agreement between the dc transport and optical dc limit resistivity values.

Following the representation used in the Mooij plot^1^ for disordered metallic systems, $R=R_0\left[ 1+\alpha _0(T-T_0)\right]$, we determine the associated TCR ($\alpha _0$) and $R_0$ (interpolated to $T_0=298$ K) values, which are listed in Table 1. Suggesting that the discontinuous nanoisland FeNi layer is non-conducting in the MLFs N1, N2, and N3, the resistivity $\rho _0$ values of the Ta layer were estimated using the equation $\rho _0=$N$R_0\gamma d$, where N is the number of layers, *d* is the nominal Ta layer thickness, and $\gamma =c/a$ is a geometrical factor determined by the ratio of the sample width to the distance between the potential contacts. The resulting $\alpha _0$ and $\rho _0$ values are listed in Table 1 and displayed in the Mooij plot coordinates in Fig. 5.

**Table 1:** TCR ($\alpha _0$) and dc resistivity ($\rho _0$) values for the Ta layer of different thickness (*d*) in the MLFs [Ta(*d*) – FeNi(*h*)]$_{\mathrm{N}}$ below the FeNi layer percolation threshold. $R_0$ is the MLF sample resistance obtained from the linear interpolation to $T_0=$ 298 K (see the text).

N	*d*	*h*	$\gamma$	$R_0$	$\alpha _0$	$\rho _0$	$\sigma _0$
	(nm)	(nm)		($\Omega$)	(ppm/K)	($\mu \Omega \cdot$cm)	($\Omega ^{-1}\cdot \hbox {cm}^{-1}$)
N1	4.6	0.52	2.25	48.2	$-552\pm 13$	499±20	2000$\pm 80$
N2	2.3	0.52	2.63	61.9	$-438\pm 5$	412$\pm 21$	2430±120
N3	1.3	0.52	2.27	51.4	$-217\pm 2$	212$\pm 13$	4720$\pm 280$
N5	2.5	1.0	2.38	31.1	$-226\pm 2$	205$\pm 10$	4880±250
N6	2.5	1.5	2.45	19.1	$-94\pm 1$	129$\pm 6$	7750$\pm 390$

We would like to note that the estimated dc conductivity values $\sigma _0$=1/$\rho _0$ (see Table 1) are in good agreement with the optical dc limit $\sigma _{1\,(\omega \rightarrow 0)}$ of the Ta layer Drude response in the MLF samples N1, N2, and N3 of $\simeq$ 2050 (4.6 nm), $\simeq$ 2550 (2.3 nm), and $\simeq$ 4650 (1.3 nm) $\Omega ^{-1}\cdot \hbox {cm}^{-1}$ (depicted in Fig. 5), resulting from our recent spectroscopic ellipsometry study^50^.

The obtained data ($\alpha _0$, $\rho _0$) for the Ta layer of different thickness in the MLFs match the range of the Mooij plot for disordered metals and alloys^1,2^. In particular, the ($\alpha _0$, $\rho _0$) values for the Ta layer in the sample N1 fall in the area around ($-500$ ppm/K, 500 $\mu \Omega \cdot$cm) on the Mooij plot, seemingly indicating its unusual transport properties. Indeed, here the dc resistivity of the Ta layer is well above the critical value $\rho ^*\sim$ 300 $\mu \Omega \cdot$cm of the MIR limit (Eq. 1) for disordered metals. The unforeseen result obtained is that the negative slope decreases in the investigated series of the MLF samples N1, N2, and N3 upon decreasing the Ta layer thickness (see Table 1 and Fig. 5). However, one would naturally expect that the absolute value of negative TCR will increase with degree of disorder in the thinner Ta layer. This is illustrated by the ($\alpha _0$, $\rho _0$) data determined for the 50- and 33-nm thick single-layer Ta films grown onto the Sitall substrate at the same rf sputtering conditions^31^ (see Fig. 5). Thus, we can conclude that the $\rho _0$ and TCR absolute values in the MLF samples N1, N2, and N3 preferentially depend on and are determined by the Ta layer thickness (and so the magnetic interaction strength). Following the Mooij rule^1^, the TCR value tends to be more negative with increasing $\rho _0$ (as demonstrated by Fig. 5).

Figure 4a–d clearly demonstrates the evolution of the dc transport in the MLF samples of the second series from the non-metallic ($d R/dT<0$ in N5 and N6) to metallic ($d R/dT>0$ in N7 and N8) character with an increase in the FeNi layer thickness from 1.0, 1.5, 2.0 to 4.0 nm. Naturally, this behaviour can be associated with the FeNi layer, which undergoes an insulator-to-metal transition due to appreciable coalescence of FeNi nanoislands in the layer with the thickness above the percolation threshold at the critical thickness $d_c$ $\simeq$ 1.5–1.8 nm^37,38^. We found that below the percolation threshold of the FeNi film the resistance variation in the MLF samples N5 and N6 can be adequately approximated by the linear dependence in the 80 – 180 K temperature range (see Fig. 4a,b) characterised by the negative slope values. Given that the discontinuous nanoisland FeNi layer is non-conducting below the percolation threshold in the samples N5 and N6, we evaluated the ($\alpha _0$, $\rho _0$) values for the Ta layer in these MLF samples (see Table 1 and Fig. 5). Here, the Ta layer dc resistivity appeared to be below the MIR limit ($\rho ^*\sim$ 300 $\mu \Omega \cdot \mathrm {cm}$) and, following the Mooij rule, their TCR values tend to be less negative (see Fig. 5). We would like to note that the *R*(*T*) dependencies shown in Figs. 3a and 4a,b indicate insignificant deviations from the linear approximations above 180 K, associated with the dc transport peculiarities, which require a more comprehensive study at elevated temperatures.

At and above the percolation threshold of the FeNi film at the critical thickness $\sim$ 1.5–1.8 nm^37,38^, the *R*(*T*) behaviour of the MLF samples N7 and N8 is determined by the cumulative contributions from the inclusive FeNi and Ta layers. The *R*(*T*) dependence of the MLF sample N7 exhibits a clearly pronounced minimum at $\sim$ 80 K (see Fig. 4c), whereas it is very weakly pronounced and shifted to the lower temperature of $\sim$ 20 K for the sample N8 (omitted on the scale of Fig. 4d). Above the minima, we observed a rise of the *R*(*T*) dependence for the samples N7 and N8 with metallic ($d R/dT>0$) character (see Fig. 4c,d). We found that here the temperature resistance variation can be well approximated by the $T^2$ dependence (omitted), which can be naturally ascribed to inelastic electron-electron scattering^14^ in the percolating and continuous FeNi layers. Here, the Ta and FeNi layers having the nominal thickness *d* and *h*, respectively, are both conducting. The resistance *R* of the MLF sample can be determined by the following relationship

2 $$\begin{aligned} \frac{1}{R\mathrm N\gamma } =\left( \frac{h}{\rho _0} \right) ^{FeNi}+\left( \frac{d}{\rho _0}\right) ^{Ta} =\left( h\sigma _0\right) ^{FeNi}+\left( d\sigma _0 \right) ^{Ta} \end{aligned}$$

expressed via the resistivities $\rho _0$ (or conductivities $\sigma _0$ = $1/\rho _0$) of the inclusive layers. However, since in Eq. (2) we have two unknown values $\rho _0^{Ta}$ and $\rho _0^{FeNi}$, it is not possible to attain the Ta layer dc conductivity properties in the MLF samples N7 and N8 via the dc transport measurements. Here, we determine the optical conductivity properties of the MLF samples N5–N8 from the spectroscopic ellipsometry study presented below.

### Spectroscopic ellipsometry of the Ta–FeNi multilayer films

In the recent publication^50^, we reported results of our wide-band spectroscopic ellispometry study (in the 0.8–8.5 eV spectral range) of the MLF samples (Ta–FeNi)$_{\mathrm{N}}$ from the first series (as schematically presented in Fig. 2a). The Ta layer complex dielectric function was obtained from the Drude-Lorentz simulations, which revealed different metallicity characters in the MLF samples N1–N3. This strongly suggests that the itinerant charge carrier response in the Ta intralayer preferentially depends on the distance between the adjacent 0.52-nm thick nanoisland FeNi layers and so determined by the magnetic interaction strength between them.

**Figure 6:**
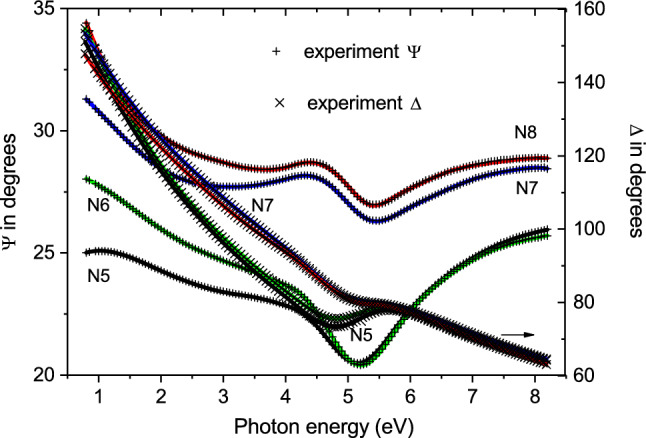
Spectroscopic ellipsometry data. Ellipsometric angles, $\Psi (\omega )$ and $\Delta (\omega )$, measured for the MLF samples N5–N8 at the angle of incidence 70$^\circ$ (displayed by the symbols). The solid lines show the fitting results using the Drude-Lorentz model (Eq. 3) in the simulation of the MLFs response.

Here, using the spectroscopic ellipsometry approach, we focused our research on the MLF samples (Ta–FeNi)$_{\mathrm{N}}$ from the second series (as schematically shown in Fig. 2b) in the limit where the FeNi layer thickness is varied across the percolation threshold around 1.5–1.8 nm^37,38^. The ellipsometric angles $\Psi (\omega )$ and $\Delta (\omega )$ were measured at room temperature at two angles of incidence of 65$^\circ$ and 70$^\circ$ (see Fig. 6). The measured ellipsometric angles $\Psi (\omega )$ and $\Delta (\omega )$ were fitted in the framework of the multilayer model [Ta(2.5 nm)–FeNi(*h*)]$_{\mathrm{11}}$–Ta(2.5 nm)/Sitall (where *h* = 1.0, 1.5, 2.0, and 4.0 nm) using the J.A. Woollam VASE software^51^. The complex dielectric function ${\tilde{\epsilon }}(\omega )=\varepsilon _1(\omega )+\mathrm {i} \varepsilon _2(\omega )$ of each layer was modeled by a Drude term, which is a zero-resonance energy Lorentz oscillator used to represent free charge carriers, and a sum of contributions from higher-energy Lorentz oscillators

3 $$\begin{aligned} {\tilde{\varepsilon }}(E\equiv \hbar \omega )=\epsilon _{\infty }-\frac{A_D}{E^2+\mathrm{i}E\gamma _D}+\sum _j\frac{A_j \gamma _jE_j}{E_j^2-E^2-\mathrm{i}E\gamma _j}, \end{aligned}$$

where $\varepsilon _{\infty }$ is the core contribution to the dielectric function. The adjustable (fitting) Drude parameters were $A_D$ (which is related to the plasma frequency $\omega _p$ via $A_D=\varepsilon _{\infty }\hbar \omega ^2_p$) and scattering rate $\gamma _D$. Each Lorentz oscillator was fitted with three adjustable parameters $E_j$, $\gamma _j$, and $A_j$ of the peak energy, the full width at half maximum, and the $\varepsilon _2$ peak height, respectively.

The Ta and FeNi layers in each MLF structure were described by different dispersion models (Eq. 3), including the Drude term and Lorentz oscillators. In the simulation of the ellipsometry data, the discontinuous nanoisland FeNi layers were represented by the effective dielectric function in the effective medium approximation (EMA). To the utilized multilayer model, the complex dielectric function spectra of the blank Sitall substrate obtained from our complementary ellipsometry measurements, were substituted. The Ta and FeNi layer thicknesses were fitted to their respective nominal values. The high quality of the fit is demonstrated by Fig. 6, where we present the measured ellipsometric angles $\Psi (\omega )$ and $\Delta (\omega )$ along with the fitting results. The quality of the fit was verified by the coincidence within the specified accuracy of less than 5% with the ellipsometric angles $\Psi (\omega )$ and $\Delta (\omega )$ measured at two angles of incidence of 65$^\circ$ and 70$^\circ$. It was verified that the simulation in the framework of the multilayer model (Ta–FeNi)$_{\mathrm{N}}$–Ta/Sitall, where the Ta–FeNi interface roughness is explicitly included, does not improve the fit (for more details, see our recently published study^50^). In particular, we expect that the Ta–FeNi interface roughness is essentially incorporated in the effective dielectric function of the nanoisland FeNi layer.

**Figure 7:**
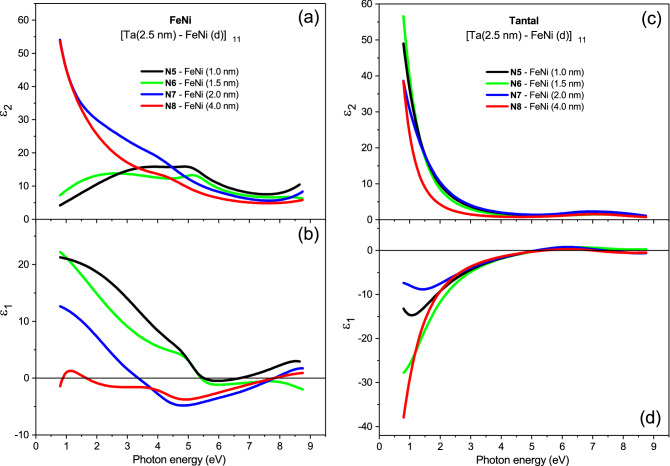
Wide-range complex dielectric function response of FeNi and Ta layers in the studied Ta–FeNi MLFs. (**a**,**c**) The $\varepsilon _2(\omega )$ and (**b**,**d**) $\varepsilon _1(\omega )$ dielectric function spectra of the FeNi and Ta layers, respectively, where the FeNi layer has different thickness of 1.0, 1.5, 2.0, and 4.0 nm in the MLF samples N5, N6, N7, and N8, and the Ta layer thickness is regular and equal to 2.5 nm (shown by solid black, green, blue, and red curves, respectively).

From the multilayer model simulations by using the dispersion model introduced by Eq. (3), the imaginary and real parts of the dielectric function spectra, $\varepsilon _2(\omega )$ and $\varepsilon _1(\omega )$, of the FeNi and Ta layers in the MLF structures N5–N8 were extracted, which are displayed in Fig. 7a–d (for more details of the dispersion analysis, see the Supplementary information). The EMA dielectric function of the FeNi layers in the MLF samples N5 and N6 was modeled by three Lorentz oscillators, while any Drude contribution was vanished out during the fit, thus notifying that the optical dc limit $\sigma ^{FeNi}_{1\,(\omega \rightarrow 0)}\approx 0$. This gives more confidence to our estimates of the Ta layer resistivity $\rho _0$ in the MLF samples N5 and N6 (see Table 1) from the present dc transport measurements relied on the guess that the discontinuous FeNi layers are non-conducting in these structures. From Fig.  7a one can clearly see that the $\varepsilon _2(\omega )$ function of the FeNi layer dramatically increases at low probed photon energies with increasing the FeNi layer thickness above the percolation threshold. Simultaneously, the $\varepsilon _1(\omega )$ function of the FeNi layer progressively decreases with increasing the FeNi layer thickness and for the 4nm-thick FeNi layer it exhibits a sharp downturn to negative values at the lowest probed photon energies (see Fig. 7b), demonstrating the metallic behaviour. The dielectric function of the FeNi layers in the MLF samples N7 and N8 was modeled by Drude resonance and four higher-energy Lorentz oscillators. According to our simulation results, we expect that the Drude resonance becomes rather narrow in the sample N8 well above the percolation transition, whereas it is quite wide at the percolation threshold in the sample N7. The resulting values of the Drude dc conductivity limit $\sigma ^{FeNi}_{1\,(\omega \rightarrow 0)}$ and scattering rate $\gamma ^{FeNi}_D$ in the MLF structures N5–N8 are given in Table 2.

**Table 2:** Optical dc limit $\sigma _{1\,(\omega \rightarrow 0)}$ and scattering rate $\gamma _D$ of the Drude resonance in the FeNi and Ta layers for the MLF samples N5–N8 [Ta(*d*=2.5 nm) – FeNi(*h*)]$_{11}$, obtained from the present spectroscopic ellipsometry study.

N	*h*	$\sigma ^{FeNi}_{1\,(\omega \rightarrow 0)}$	$\gamma ^{FeNi}_D$	$\sigma ^{Ta}_{1\,(\omega \rightarrow 0)}$	$\gamma ^{Ta}_D$
	(nm)	($\Omega ^{-1}\cdot \hbox {cm}^{-1}$)	(eV)	($\Omega ^{-1}\cdot \hbox {cm}^{-1}$)	(eV)
N5	1.0	0	–	4400	1.29
N6	1.5	0	–	7300	1.00
N7	2.0	5290	3.64	4360	0.86
N8	4.0	9390	0.39	8450	0.78

From Fig. 7c,d one can follow the trends of the foreseen behaviour in the complex dielectric function spectra of the 2.5-nm thick Ta intralayer accompanying the insulator-to-metal transition within the FeNi layer, resulting from the current Drude-Lorentz model simulations of the MLF samples N5–N8. Note that the metallicity character of the Ta layer changes in the MLF sample series N5–N8 across the FeNi film percolation transition, although not sequentially. Indeed, the displayed $\varepsilon _2(\omega )$ and $\varepsilon _1(\omega )$ spectra indicate that, in agreement with the dc transport measurements (see Table 1 and Fig. 5) the Ta layer in the sample N6 reveals better metallicity properties than that in the sample N5. The dielectric function response of the Ta layer in the sample N7 suggests its poorer metallicity properties than in the sample N6, whereas the best metallicity properties in the whole MLF sample series N5–N8 are exhibited by the Ta layer in the sample N8.

**Figure 8:**
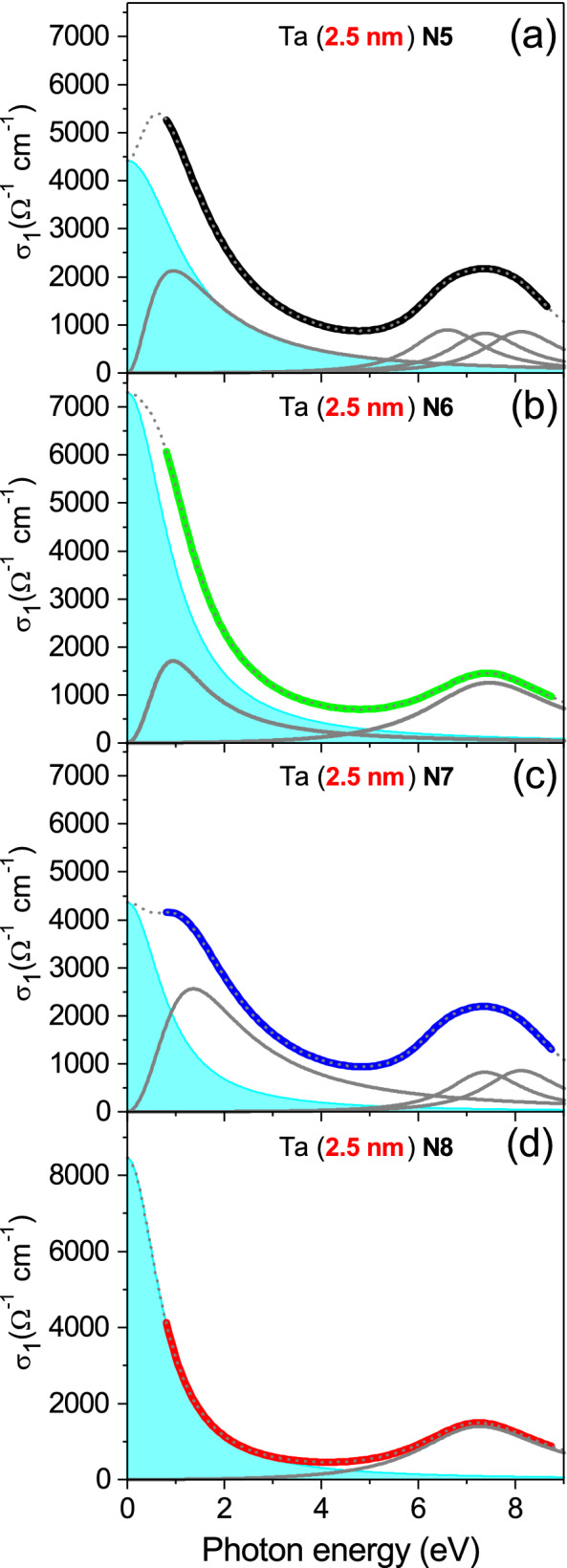
Wide-range optical conductivity of the Ta layer in the MLFs Ta–FeNi. (**a**–**d**) The Ta layer optical conductivity $\sigma _1(\omega )=\frac{1}{4\pi }\omega \varepsilon _2(\omega )$ in the MLF samples N5, N6, N7, and N8 shown by solid black, green, blue, and red curves, respectively. The contributions from the Ta layer Drude term is shown by the cyan shaded area and from the Lorentz oscillators by solid gray lines. The agreement of the dispersion analysis with the $\sigma _1(\omega )$ dependence is demonstrated by the summary Drude-Lorentz contribution, shown by dotted lines.

Figure 8a–d displays the evolution of the Ta intralayer optical conductivity $\sigma _1(\omega )$ = $\frac{1}{4\pi }\omega \varepsilon _2(\omega )$ upon increasing the FeNi layer thickness *h* across the percolation threshold in the MLF structures [Ta(2.5 nm)–FeNi(*h*)]$_{11}$ for the samples N5–N8, respectively. Here, the contributions from the Drude term and Lorentz oscillators resulting from the multilayer model simulations using Eq. (3) are explicitly demonstrated. The corresponding values of the optical Drude conductivity limit $\sigma ^{Ta}_{1\,(\omega \rightarrow 0)}$ and scattering rate $\gamma ^{Ta}_D$ are presented in Table 2. From Fig. 8a–d one can see that the Ta layer optical conductivity $\sigma _1(\omega )$ in the MLF structures N5–N8 can be well described in the framework of the elaborated Drude-Lorentz model.

## Discussion

Let us follow the trends occurring in the Ta layer optical conductivity for the samples N2^50^, the 33-nm thick single Ta layer^30,31^, N5, and N6, which span a major part of the ($\alpha _0$, $\rho _0$) Mooij plot related to the negative TCR values (see Fig. 5). According to the results obtained from the dc transport study (see Table 1), the Ta layer in the sample N6 (Ta = 2.5 nm, FeNi = 1.5 nm) exhibits the best dc conductivity properties with $\alpha _0\sim -$ 95 ppm/K and $\rho _0\sim$124 $\mu \Omega \cdot \hbox {cm}^{-1}$. For the chosen sample series, the Ta layer in the sample N2 (Ta = 2.3 nm, FeNi = 0.52 nm) demonstrates the worst conductivity properties with $\alpha _0\sim -$ 438 ppm/K and $\rho _0\sim$ 412 $\mu \Omega \cdot \hbox {cm}^{-1}$. The 33-nm single-layer Ta film displays the dc conductivity properties peculiar to the MIR limit. Indeed, according to the dispersion analysis using Eq. (3), the Ta layer low-energy response is dominated by the Drude term demonstrating the optical dc conductivity limit $\sigma ^{Ta}_{1\,(\omega \rightarrow 0)}\sim$ 3300 $\Omega ^{-1}\cdot \hbox {cm}^{-1}$ (in good agreement with Eq. 1) and the intense low-energy Lorentz band peaking at 2.2 eV^30^. What are the main trends observed in the optical conductivity spectra of these sample on moving along the ($\alpha _0$, $\rho _0$) line (see Fig. 5) in the opposite directions from the MIR limit? Analyzing the optical conductivity spectra shown in Fig. 8a–d and in our previous papers (see Fig. 4d^30,50^) one can see the following systematic changes (i) the Drude dc conductivity decreases below the MIR limit and the low-energy band shifts to a higher energy of 2.7 eV in the sample N2, (ii) the Drude dc conductivity increases above the MIR limit and the low-energy band shifts to the lower energy about 1 eV in the samples N5 and N6, and (iii) the intensity of the low-energy band decreases on the conductivity enhancement in the sample N6. According to our simulations, the best metallicity properties among the whole MLF sample series N5–N8 are demonstrated by the Ta layer in the sample N8 (see Table 2 and Fig. 8d), where the low-energy band completely disappears from the optical conductivity spectrum.

Near the percolation transition within the FeNi layers strong magnetic dipole-dipole and exchange interactions between the FM nanoislands become relevant. It was shown that in quasi-2D systems of FM nanoparticles collective superferromagnetic (SFM) states can exist in their self-assembled local arrangements (clusters) at comparatively high temperatures^36,39,40^. We suppose that in here GMR-like case is realized, where the interlayer coupling between two neighboring FeNi layers is driven by the indirect exchange interactions via the itinerant charge carriers of the Ta spacer layer. Their kinetic energy enhancement in the regime of strong FM correlations between giant magnetic moments of FeNi nanoislands (similar to the double-exchange (DE) model used in the description of the colossal magnetoresistance (CMR) effect) may lead to the delocalisation of electrons from the associated localised electronic states. As a result, the Drude contribution increases implying the delocalisation of charge carriers simultaneously with the intensity decrease of the low-energy excitation band. According to the obtained results, this may lead to the substantial Ta layer resistivity decrease of about 60% (normalized to the MIR limit value $\rho ^*\sim$ 300 $\mu \Omega \cdot$cm), introduced by the magnetic interaction between the FeNi layers.

Another paradigm is represented by the MLF sample N1, where the 0.52-nm thick FeNi layer is far from the percolation transition and the FeNi layers are well separated from each other (by 4.6 nm). Earlier, we established that ultrathin monolayers of FeNi nanoislands exhibit superparamagnetic-like (SPM) behaviour at room temperature, associated with their giant magnetic moments of 10$^3$–10$^5$ $\mu _{\mathrm{B}}$^36,40^. In the MLF sample N1, incorporating such FeNi nanoisland layers, the Drude dc conductivity drops well below the MIR limit (see Table 1) and the low-energy band at 2 eV becomes essentially suppressed, while the pronounced increase in the higher-energy band around 6–8 eV is observed (see Fig. 4e from our previous article^50^), which can be associated with new localised electronic states. According to the obtained results, these new localisation phenomena introduced by an additional strong magnetic disorder and long-range many-body interactions between giant magnetic moments of FeNi nanoislands via the remaining free itinerant charge carriers of a disordered metal by means of RKKY-type indirect exchange, lead to the additional Ta layer resistivity increase (normalized to the MIR limit value $\rho ^*\sim$ 300 $\mu \Omega \cdot$cm) up to 60%.

To shed light on our experimental findings, below we present a simple model of giant magnetic moments (superspins $\mathbf{S} _i=(S_{xi},S_{yi},S_{zi})$) of FM FeNi nanoislands interacting via indirect exchange mediated by conduction electrons of Ta layers. This model can be described by the following Hamiltonian

4 $$\begin{aligned} H_0 = \sum \limits _{ i,j} J( k_{\mathrm{F}} r_{ij})(\mathbf{S} _i \cdot \mathbf{S} _j), \end{aligned}$$

where $k_{\mathrm{F}}$ is the Fermi momentum of the Fermi sea associated with the conduction electrons of the Ta nanolayer, and $J( k_{\mathrm{F}} r_{ij})$ is responsible for the interaction between the superspins $\mathbf{S} _i$ and $\mathbf{S} _j$ separated by the distance $r_{ij}$, which occurs due to Friedel-like oscillations of electron spin density induced by the spin polarization of an electron cloud nearby a FM FeNi nanoisland. Such polarization arises due to exchange forces between localised magnetic moments of the FM FeNi nanoislands and mobile conduction electrons of the Ta nanolayer. The suggested interaction model has a certain similarity to the RKKY interaction^33–35^. The system under study has a quasi-two-dimensional character implied by the structure of Ta/FeNi nanolayers. Due to this quasi-two-dimensionality, the long-range interaction between superspins $\mathbf{S} _i$ and $\mathbf{S} _j$ of the FeNi nanoislands separated by the distance *x* takes the form^52^

5 $$\begin{aligned} J(x) \sim \frac{n^2 J^2}{E_F}\frac{\sin {x}}{x^2}, \end{aligned}$$

where *n* is the electron density per Ta atom, *J* is the exchange interaction between electron spins of the Ta subsystem and superspins of FeNi nanoislands.

In our approach, we used second-order perturbation theory to describe an indirect exchange coupling whereby the spin of one Fe or Ni atom of a FeNi nanoisland interacts with a conduction electron of Ta, which then interacts with another spin of different FeNi nanoisland. Making summation over all atoms of one FeNi nanoisland, we will have interaction of the Ta conduction electron with the superspin of this FeNi nanoisland. Doing the same summation for the second FeNi nanoisland, we can find out a correlation energy between the two superspins associated with two different magnetic FeNi nanoislands. Thereby, the superspins produce a pronounced effect on the Fermi sea of electrons in the Ta nanolayer. When the Ta layer thickness is fixed (as for the Ta–FeNi MLFs from the second series), and so the distance between neighboring FeNi layers is fixed at 2.5 nm, the interaction in Eq. (4) will be ferromagnetic^53^ and will have a certain similarity to the double-exchange (DE) model, which is used, in particular, in description of the transport mechanism and optical conductivity properties of colossal magnetoresistive (CMR) manganites^54,55^.

When the FeNi layer is discontinuous and represented by randomly distributed distant FM FeNi nanoislands, this interaction will oscillate from ferromagnetic to the antiferromagnetic one with the period, which is very small, about a few nanometers. Therefore, the strong interaction between superspins oscillates between ferromagnetic and antiferromagnetic types as a function of the distance between the FeNi nanoislands. Note that even the size of these islands can be comparable with the period of such oscillations. This creates a strong frustration in the system impeding finding its ground state. As a result, the system is slowly migrating from one metastable state to another reminding the situation characteristic of spin and orbital glass systems^56^. Such frustrations and strong interactions between superspins can lead to some kind of a non-ergodic state.

To summarize, using dc transport and wide-band (0.8–8.5 eV) spectroscopic ellipsometry experimental techniques, we investigated the conductivity properties of the Ta intralayer inside the MLF structures (Ta–FeNi)$_{\mathrm{N}}$ in two limits of the magnetic layer thickness (i) when the FeNi layer has a discontinuous nanoisland structure and (ii) when the FeNi layer thickness is increasing across the percolation threshold. We found that for the nanoisland structure of the FeNi layer the Ta layer dc resistance temperature variation can be adequately approximated by the linear dependence in the 80 – 180 K temperature range characterised by the negative TCR ($\alpha _0$) values peculiar of strongly disordered metallic systems^18^. Following the Mooij rule^1^, the determined values of $\alpha _0$ and dc resistivity $\rho _0$ fit the linear dependence, where the TCR values tend to be more negative with increasing $\rho _0$ (see Fig. 5). We discovered that the dc transport of the Ta layer strongly depends on the structural properties of the FeNi layers around the metal-insulator percolation transition, which, in turn, determine their magnetic properties^40^ and, thereby, the stregth of magnetic coupling between neighboring FeNi layers mediated by the RKKY-type interaction. The entanglement of the RKKY-type interaction with the itinerant electrons of the Ta spacer lies in the root of the observed Mooij correlations driven by the interplay between the strength of the magnetic interaction and localisation of electons in the disordered Ta–nanoisland FeNi MLF structures. According to our results, this may lead to a substantial Ta layer resistivity decrease of about 60% (normalized to the MIR limit value $\rho ^*\sim$ 300 $\mu \Omega \cdot$cm). From the multilayer model simulations, we extracted the Ta and FeNi layer dielectric function response in the (Ta–FeNi)$_{\mathrm{N}}$ MLFs. Near the MIR limit the Ta layer dielectric function is represented by the Drude resonance due to the Ta layer free charge carriers superimposed with the low-energy excitations at around 2–4 eV. The low-energy excitaions appear in evidence of strong electron correlations in the systems with strong electron correlations^25–28^. We suggest that electronic correlations accompany the localisation of electrons in clusters of electronic inhomogeneities. We found that when the dc conductivity and the optical Drude dc conductivity limit consistently increase above the MIR regime the low-energy excitations exhibit the red shift to about 1 eV, their intensity decreases, and finally their fingerprints dissappear when the FeNi layer has the thickness well above the percolation threshold. The observed behaviour signals progressive delocalisation of electrons with increasing the indirect exchange coupling strength.

Thus, we discovered that the Ta layer resistivity critically depends on the properties of the FeNi layer, when its morphology changes from the discontinuous nanoisland to continuous structure across the percolation threshold, where the TCR and $\rho _0$ values obey the Mooij correlations. In the optical conductivity spectra, while approaching the MIR limit, the Drude contribution of charge carriers decreases, signaling the enhanced localization effects, which are accompanied by the appearance of the low-energy optical bands in the spectral range (1–2 eV) characteristic of electronic correlations.

Moreover, we discovered that when the FeNi layer is represented by distant randomly distributed giant magnetic moments of magnetic FeNi nanoislands the additional localisation effects where the Ta layer normalized dc conductivity falls down below the MIR limit by about 60% take place. The discovered phenomenon, which can be associated with a large-scale fluctuating potential of magnetic origin and lead to non-ergodicity and purely quantum localisation effects^45–49^, need to be further challenged theoretically and experimentally. The obtained results demonstrate the advances of the used spectroscopic ellipsometry approach, which allowed us to extract the dielectric function response of each inclusive layer within the ultrathin MLF structures, and must be essential for understanding the physics of the Anderson localisation phenomena in magnetic multilayers and from the application view of the GMR effect.

## Methods

The MLFs (Ta–FeNi)$_{\mathrm{N}}$–Ta were grown by alternating rf sputtering from 99.95% pure Ta and $\hbox {Fe}_{{21}}\hbox {Ni}_{{79}}$ targets onto insulating Sitall-glass substrates. The actual substrate temperature during deposition was 80 $^\circ$C. The base pressure in the chamber was 2$\times$10$^{-6}$ Torr, and Ar gas flow with a pressure of 6$\times$10$^{-4}$ Torr was used for the sputtering process. The Ta and FeNi layer nominal thickness was determined by the deposition time, and the deposition rate was about 0.067 nm/s. The sputtered MLFs were characterised by X-ray diffraction (XRD) analysis. The periodicity of the grown MLFs (Ta – FeNi)$_{\mathrm{N}}$ was characterised by low-angle X-ray reflectometry. The X-ray measurements were carried out on a Bede 200 Goniometer operating at 55 kV and 300 mA with Cu $\hbox {K}_\alpha$ radiation $\lambda$ = 0.154 nm produced by an X-ray Generator with a rotating Rigaku RU 300 anode (for more details, see the Supplementary information to this paper).

The microstructure of the MLFs (Ta–FeNi)$_{\mathrm{N}}$ was examined on a Titan 80-300 scanning/transmission electron microscope (STEM) (FEI, US) operating at the accelerating voltage 300 kV and equipped with the spherical aberration corrector of an electron probe in the bright- and dark-field regimes. In the dark-field imaging a high-angle annular dark-field (HAADF) detector (Fischione, US) registering large-angle scattered electrons was used. The cross-section specimens were prepared by the standard focus ion beam procedure on Versa 3D and Helios NanoLab 600i (Thermo Fisher Scientific, US) dual beam microscopes. The instruments were equipped with an Omniprobe micromanipulator (Omniprobe, US) and gas injection systems for Pt and W deposition. At the first stage, 2-$\mu$m thick W capping layer was formed at the sample surface to protect from possible damages. The cuts were performed using a 30 keV $\hbox {Ga}^+$ ion beam. To remove the amorphous layer at the final stage of the cutting, the $\hbox {Ga}^+$ ion beam energy was reduced to 2 keV. Energy dispersive X-ray (EDX) microanalysis was performed using a spectrometer (Phoenix System, EDAX, US). (for more details, see the Supplementary information to this paper).

## Experimental approach

The dc resistance of the rf-sputtered MLFs was measured in a standard four-probe configuration in the linear I−V regime using lock-in amplifiers (MFLI, Zurich instruments) and a voltage controlled current source (CS580, Stanford Research Systems). Ohmic contacts to the MLF samples were made by soldering. The samples were mounted on a cold finger inside the home-made insert and evacuated to a low-pressure environment at room temperature. The dc resistance was measured on heating in a wide temperature range of 5–250 K. To avoid noticeable temperature delay during the measurements, the temperature variation rate was adjusted at 3.5 K/min.

In our complementary optical study, we used spectroscopic ellipsometry approach. The grown MLF samples (Ta–FeNi)$_{\mathrm{N}}$–Ta/Sitall were measured by spectroscopic ellipsometry in a wide photon energy range of 0.8–8.5 eV with a J.A. Woollam VUV-VASE Gen II spectroscopic ellipsometer. The ellipsometry probes were obtained on the prepared MLF samples at two angles of incidence of 65$^\circ$ and 70$^\circ$ at room temperature, as well as on the blank Sitall substrate.

## Supplementary information

Supplementary Information
