# Supplementary material for: Control of Mooij correlations at the nanoscale in the disordered metallic Ta–nanoisland FeNi multilayers
Source: Sci Rep. 2020 Dec 3;10:21172. doi: 10.1038/s41598-020-78185-6 (PMC7713312; doi:10.1038/s41598-020-78185-6)
Supplement: Supplementary file 1 — Supplementary Information [file 41598_2020_78185_MOESM1_ESM.pdf]

Supplementary online information for the article entitled

Control of Mooij correlations at the nanoscale in the disordered metallic Ta – nanoisland FeNi multilayers

by N. N. Kovaleva, F. V. Kusmartsev, A. B. Mekhiya, I. N. Trunkin, D. Chvostova, A. B. Davydov, L. N. Oveshnikov, O. Pacherova, I. A. Sherstnev, A. Kusmartseva, K. I. Kugel, A. Dejneka, F. A. Pudonin, Y. Luo, & B. A. Aronzon

XRD and STEM characterization of Ta–FeNi MLFs

Figure S1(a,b) shows the superimposed XRD patterns of the blank Sitall substrate and that of the MLF samples N1 and N8, respectively. The analysis of the XRD pattern showed that the blank Sitall substrate is represented by the TiO_2 rutile phase [14]. No sharp peaks characterizing the crystalline β-Ta phase, neither that of the α-Ta cubic phase [16] were clearly seen in the XRD pattern of the MLF sample N1. In Fig. 1(a) we also present the XRD pattern of the 70-nm thick Ta layer grown on the Sitall substrate under the same rf-sputtering conditions, where the peak with 2θ at 37.8° is clearly observable, which may show a broadened shape of the sum of the peaks (202) and (212) of β-Ta, indicating that it is strongly disordered and rather represented by a mixture of the amorphous and fine-crystalline phases [15]. To some extent, this may be relevant for the Ta layers in the MLF sample N1, displaying a low XRD intensity increase relative to the Sitall substrate background nearly in the same range of scattering angles. The XRD analysis did not reveal any clear traces of Ta oxides (such as TaO_2 and Ta_2O_5) in the Ta layers. By contrast, the XRD pattern of the MLF N8 showed the clearly pronounced (111) peak associated with the face-centered cubic (FCC) polycrystal structure with a cell parameter of 3.54 ± 0.03 Å, identical to the lattice constant of the bulk $Ni_{80}Fe_{20}$ random alloy [S1].

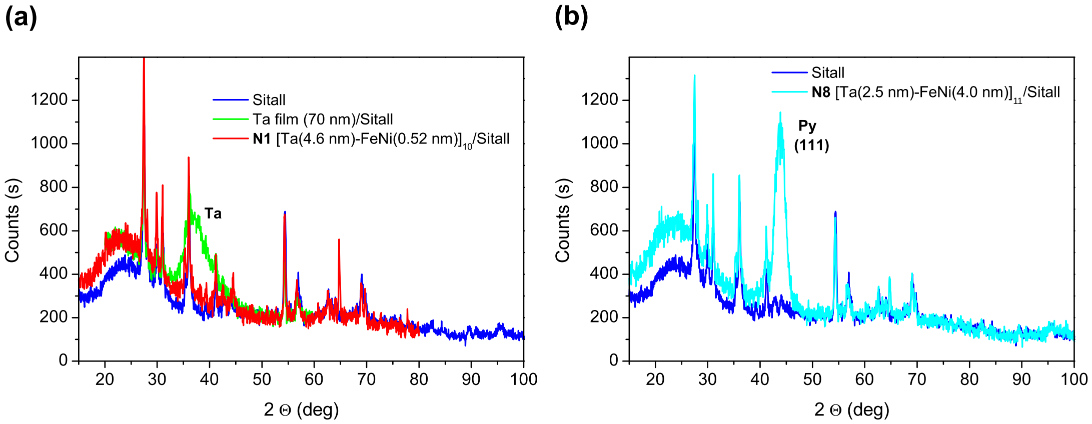

FIG. S1. **X-ray diffraction characterization of the grown Ta–FeNi MLFs** The superimposed XRD patterns of the blank Sitall substrate (blue curve) and the MLF samples $(Ta-FeNi)_N-Ta$/Sitall substrate (a) N1 (red curve) and (b) N8 (cyan curve). The XRD pattern of the 70-nm thick Ta film grown on the Sitall substrate under the same rf-sputtering conditions is shown in (a) by the green curve.

The X-ray reflection from the representative MLF samples from the first and second series (N1 and N8) exhibits superlattice peaks (Kiessig's oscillations), as one can see from Fig. 2(e,f), respectively, indicating a periodic compositional modulation along the film growth direction. Usually, the better interface quality, the larger interval of angles for which the oscillations can be observed. The observed Kiessig's oscillations resolved up to the third order give evidence of the relatively small interface roughness. For the MLF structure analysis, we used the Bruker Diffrac plus Leptos computational simulation, based on the theory of X-ray reflectometry. The estimated thickness of the Ta (FeNi) layer equal to 4.86 (0.96) and 2.42 (3.48) nm is in good agreement with nominal Ta (FeNi) thicknesses of 4.6 (0.52) and 2.5 (4.0) nm in the MLFs N1 and N8, respectively. The estimated density of the Ta (FeNi) layer was 14.2 (4.1) and 15.1 (9.0) g/cm^3 in the samples N1 and N8, which is in satisfactory agreement with a bulk Ta density of 16.6 g/cm^3. The estimated density of the discontinuous nanoisland FeNi layer in the sample N1 is less than a bulk FeNi density of 8.62 g/cm^3. However, that of the continuous FeNi layer in the samples N8 is in a reasonable agreement with the bulk FeNi density. The estimated roughness for the Ta and FeNi interfaces in the MLFs N1 and N8 was about 0.6

and 0.97 nm, respectively. For more details on the x-ray reflectivity study of the MLF samples N1, N2, and N3, see our recent publication [40].

EDX spectroscopy combined with STEM was used to verify the elemental composition of the layers in the studied MLFs $(Ta–FeNi)_N$. A closer inspection of the FeNi layer areas in the specimen N1 (see Fig. S2(a)) revealed that these layers are not uniform and not continuous, which can be illustrated by the STEM intensity profile (see Fig. S2(b)) for the corresponding region (marked with a rectangle in Fig. S2(a)). The distinct peaks in the intensity profile are related to the presence of FeNi nanoislands 3–5 nm in diameter and 0.4–0.8 nm in height. According to the EDX results, the FeNi layer contains up to 6% Fe and up to 23% Ni, which is consistent with the used $Fe_{21}Ni_{79}$ permalloy target composition. The EDX results for the areas outside the FeNi layers representing the Ta layer composition suggest 100 to 92% of Ta with a possible low contribution from 0 to 8% Ni. The HAADF STEM image from the Ta and FeNi layers in the cross section image of the sample N8 is shown in Fig. S3. According to the EDX results, the FeNi layers in this sample contain from 5 to 6% Fe, from 79 to 85% Ni, and from 8 to 13% Ta. The composition of the Ta layers varied from 92 to 100% Ta, which may incorporate up to 8% Ni. The Ta layer looks rather disordered, represented by a mixture of the amorphous and fine-crystalline phases. In the FeNi layers, we observed the formation of the polycrystalline FCC structure of $Ni_{80}Fe_{20}$ [S1] supporting the results of the XRD analysis presented in Fig. S1(b).

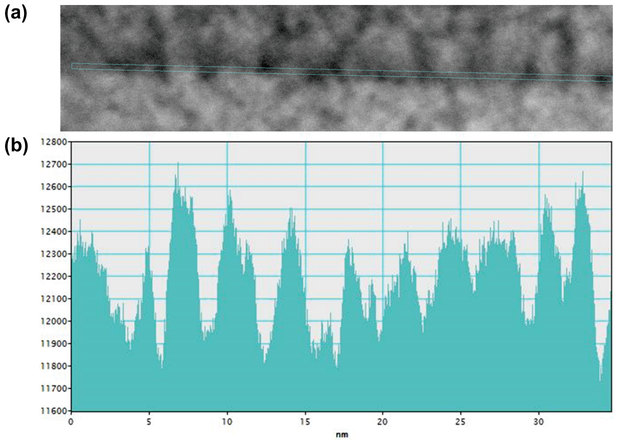

FIG. S2. **HAADF STEM study** of the 0.52 nm FeNi layer in the sample N1. (a) Focused image of the FeNi layer cross-section and (b) the corresponding linear intensity profile in the marked rectangle area.

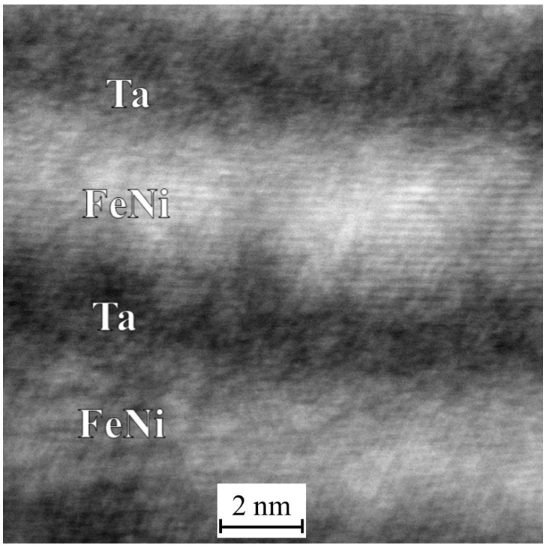

FIG. S3. **Bright-field STEM image** from the FeNi (light gray color) and Ta (dark gray color) layers in the cross section image of the sample N8.

TABLE I: **Drude-Lorentz analysis.** Parameters of the Drude band and Lorentzian oscillators for the Ta layer resulting from the fit of the complex dielectric response (Eq. (1)) to the measured ellipsometric angles $\Psi(\omega)$ and $\Delta(\omega)$ in the framework of the multilayer model [Ta(d=2.5 nm) – FeNi(h)]$_{11}$-Ta(2.5 nm)/Sitall using the J.A. Woollam VASE softaware [41].

	parameters	N5($h = 1.0$ nm)	N6($h = 1.5$ nm)	N7($h = 2.0$ nm)	N8($h = 4.0$ nm)
	ε_1	1.32	1.72	1.53	0.73
Drude	A_D	32.89	54.34	32.47	62.93
	γ_D (eV)	1.29	0.86	0.86	0.78
interband	E_1	0.95	0.94	1.36	7.27
transitions	A_1	16.67	13.48	14.01	1.43
	γ_1	2.0	1.53	2.33	3.16
	E_2	6.60	7.46	6.60	
	A_2	1.01	1.25	1.01	
	γ_2	1.83	3.24	1.83	
	E_3	7.38		7.38	
	A_3	0.83		0.83	
	γ_3	1.89		1.89	
	E_4			7.38	
	A_4			0.83	
	γ_4			1.89	

Drude-Lorentz analysis of the dielectric function spectra

The ellipsometric angles $\Psi(\omega)$ and $\Delta(\omega)$ were measured at room temperature at two angles of incidence of 65° and 70° (see Fig. 6). The measured ellipsometric angles $\Psi(\omega)$ and $\Delta(\omega)$ were fitted in the framework of the multilayer model [Ta(2.5 nm)–FeNi(h)]$_{11}$–Ta(2.5 nm)/Sitall (where $h = 1.0, 1.5, 2.0,$ and 4.0 nm) using the J.A. Woollam VASE software [41]. The complex dielectric function $\tilde{\epsilon}(\omega) = \varepsilon_1(\omega) + \mathrm{i}\varepsilon_2(\omega)$ of each layer was modeled by a Drude term, which is a zero-resonance energy Lorentz oscillator used to represent free charge carriers, and a sum of contributions from higher-energy Lorentz oscillators

$$\tilde{\varepsilon}(E \equiv \hbar\omega) = \epsilon_\infty - \frac{A_D}{E^2 + \mathrm{i}E\gamma_D} + \sum_j \frac{A_j \gamma_j E_j}{E_j^2 - E^2 - \mathrm{i}E\gamma_j}, \tag{1}$$

where ε_∞ is the core contribution to the dielectric function. The adjustable (fitting) Drude parameters were A_D (which is related to the plasma frequency ω_p via $A_D = \varepsilon_\infty \hbar\omega_p^2$) and scattering rate γ_D. Each Lorentz oscillator was fitted with three adjustable parameters E_j, γ_j, and A_j of the peak energy, the full width at half maximum, and the ε_2 peak height, respectively. The results of the dispersion analysis of the Ta–FeNi MLFs N5, N6, N7, and N8 are summarized in Table S1.

Supplementary references

[S1] Bozorth, R. M. *Ferromagnetism*; Princeton/Van Nostrand-Reinhold: New York, 1956.
